# An *ACOT4* Multi-Nucleotide Variant Is Associated with Cardiovascular Risk in Norfolk Island and UK Biobank Cohorts

**DOI:** 10.3390/genes17020205

**Published:** 2026-02-09

**Authors:** Jacob W. I. Meyjes-Brown, Heidi G. Sutherland, Kim Ngan Tran, Miles C. Benton, Rod A. Lea, Lyn R. Griffiths

**Affiliations:** 1 Genomics Research Centre, Centre for Genomics and Personalised Health, School of Biomedical Sciences, Queensland University of Technology, Brisbane City 4000, QLD, Australia; 2 Oxford Nanopore Technologies PLC, Singapore 138667, Singapore

**Keywords:** blood pressure, HDL cholesterol, ACOT4, genetic variant, Norfolk Island population isolate, UK Biobank

## Abstract

Background: Cholesterol imbalances and elevated blood pressure (BP) are closely interrelated risk factors for cardiovascular disease (CVD) and are subject to genetic influences. We sought to identify novel associations between candidate genetic coding variants and CVD traits in our isolated study cohort and validate them in a general population cohort. Methods: We leveraged the population genetic features of the Norfolk Island Health Study (NIHS, *n* = 601), to identify candidate functional variants which were analysed for association with CVD and metabolic syndrome traits. We followed up suggestive variant-trait associations in the 2022 release of UK Biobank whole exome data (*n* = 200,625). Results: We identified a novel ten-base-pair in-frame missense multi-nucleotide variant (MNV), tagged by rs35724886, in the lipid metabolism gene *ACOT4*, which was associated with cholesterol levels and blood pressure. The MNV was associated with a lower incidence of ‘elevated BP’—systolic BP ≥ 130 mmHg or diastolic BP ≥ 80 mmHg—(OR: 0.70; 95% CI: 0.51, 0.97; *p* = 0.03), and higher total cholesterol levels (β = 0.08; *p* = 0.04) in the NIHS. Validation in the UK Biobank revealed consistent associations between the MNV (proxied by rs35725886) and lower incidence of ‘elevated BP’ (*p* = 0.0001), higher total cholesterol (*p* = 0.01), and reduced use of medication for managing blood pressure (*p* = 1.8 × 10^−6^) and cholesterol (*p* = 0.002). Structural modelling and in-silico predictions suggested that the MNV introduced destabilising changes in the ACOT4 protein, likely influencing peroxisomal lipid metabolism pathways critical to CVD risk. Conclusions: This study identified a coding MNV with potential implications for understanding the genetic regulation of lipid metabolism and its impact on cardiovascular health.

## 1. Introduction

Cardiovascular disease (CVD) remains a leading cause of mortality worldwide, with cholesterol dysregulation as a major risk factor [1]. Circulating cholesterol bound to lipoproteins in the form of LDL is known to cause vascular damage, inflammation, and the buildup of atherosclerotic plaques [2]. The HDL form is inversely correlated with CVD risk at low levels and was thought to be protective against these effects due to its putative role in reverse cholesterol transport from peripheral tissues—including plaque foam cells—to the liver, ultimately for excretion [2]. It should be noted, however, that inconsistencies in this correlation at higher concentrations, and the precise complex composition of the heterogeneous mixture of particles included in the class “HDL” have brought this relationship under renewed scrutiny [3,4]. It is well known that lifestyle factors such as diet, exercise, and tobacco use, contribute to CVD risk. Nevertheless, genetic variation plays a significant role, with heritability estimates in the region of 75% for cholesterol [5].

We sought to identify novel associations between candidate genetic coding variants and CVD traits in our isolated study cohort: the Norfolk Island Health Study. We then went on to validate these associations in a general population cohort: the UK Biobank. Exploratory analysis of whole-genome sequence (WGS) data from the NIHS indicated a candidate coding variant suggestively associated with cholesterol and blood pressure measures.

The Norfolk Island Health Study (NIHS, *n* = 601) was established in 2000 to investigate genetic risk factors for complex diseases [6,7,8]. This cohort is derived from a small admixed founder group of male European and female Polynesian ancestry that originated from the 1789 mutiny on the HMS *Bounty*, and has remained genetically distinct due to historical isolation. The Norfolk Island population exhibits a higher prevalence of metabolic conditions than the general Australian population [9,10]. For example, 51% of all NIHS participants, and 56% of those aged 65 years and over had blood cholesterol measures exceeding the high cholesterol threshold value of 5.5 mmol/L. In comparison, 26% of Australians 65 years and over reported a high cholesterol diagnosis in the 2022 Australian National Health Survey [11]. This, along with their unique population genetic characteristics makes Norfolk Island a suitable cohort for studying genetic determinants of cardiovascular risk factors.

Coding variant rs35724886 in peroxisomal lipid metabolism gene Acyl-CoA Thioesterase 4, *ACOT4* NM_152331.4:c.560C > A (p.Ala187Asp), has been associated with vascular disease [12] and has been identified as a significant metabolic quantitative trait locus by untargeted genomic and metabolomic profiling [13]. *ACOT4* has also been considered as a treatment target in non-alcoholic fatty liver disease [14]. The growing recognition of *ACOT4*′s importance in metabolic health and the results from our pilot analysis prompted us to perform an *ACOT4* gene sequencing study with respect to cholesterol levels and other related metabolic traits in the well-characterised Norfolk Island genetic isolate.

In this study we utilise the Norfolk Island cohort to show that the rs35724886 SNP is part of a ten-base-pair in-frame coding multinucleotide variant (MNV) present in both founder and non-founder NIHS participants, and is associated with improved cholesterol and blood pressure features. Independent validation of these results in a general population was undertaken using the UK Biobank’s (UKBB) whole exome sequence (WES) data, released in 2022 (*n* = 200,625) [15].

## 2. Materials and Methods

### 2.1. Norfolk Island Health Study Background

#### 2.1.1. Informed Consent Statement

Informed consent for participation was obtained from all subjects involved in the study.

#### 2.1.2. NIHS Specimen and Data Collection

The NIHS is an ongoing research program conducted in collaboration with the people of Norfolk Island. The initial collection in 2000 is described in detail by Bellis et al. [6].

Briefly, volunteers from the Island (*n* = 601) were recruited via radio and newspaper announcements; gave informed consent prior to participation; answered a questionnaire on lifestyle, medical and family history; and provided anthropometric measurements and blood samples. The blood samples were stored at −80 °C, and DNA was extracted by standard salting out. Blood chemistry measurements were performed by Queensland Medical Laboratories (Southport, QLD, Australia).

Ethical approval was granted prior to the commencement of the study by the Griffith University Human Research Ethics Committee (approval no: 1300000485). Ethics approval and management of the NIHS have since been transferred to Queensland University of Technology (approval no: 1600000464; approval date 20 April 2016).

#### 2.1.3. NIHS Whole Genome Sequencing, Identification of MNV, Exploratory Analysis

From the core related pedigree [8], 108 participants related to the population founders were selected for whole genome sequencing, which was performed by the Garvan Institute (Darlinghurst, NSW, Australia) using the Illumina HiSeq X Ten platform(Illumina, Inc., San Diego, CA, USA).

Each sample reached a minimum average sequencing depth of 25×. Quality control of raw data was performed in-house. Low-quality bases (Phred score < 20) and adapter sequences were trimmed with Trimmomatic v0.29 [16], with trimmed reads assessed for quality with FastQC v0.11.9 [17]. Reads were aligned to GRCh38/hg38 with BWA-MEM v0.7.17 [18]. Duplicate reads were marked using Picard Toolkit v2.23.8 [19], and base quality score recalibration and indel realignment were performed with GATK v4.2.0 [20]. Variant calling was performed using the GATK v4.2.0 HaplotypeCaller in gVCF mode for each sample, followed by joint genotyping using GenotypeGVCFs. Hard filters were applied following GATK best practices: variants with quality by depth (QD) < 2.0, Fisher strand bias (FS) > 60.0, or mapping quality (MQ) < 40.0 were excluded. Variants failing to meet these criteria or with genotype quality (GQ) < 20 or depth (DP) < 10 were removed. Variants were tested for deviation from the Hardy–Weinberg equilibrium (HWE) using PLINK1.9 [21], and those with *p* < 10^−6^ were discarded.

SNPs with existing RefSeq numbers were filtered to select variants that are globally rare but with elevated minor allele frequency (MAF) in the WGS group. We used MAF thresholds of <1% in gnomAD’s global category, and >5% in the NI WGS group.

SNPs passing this criterion were then evaluated with a suite of bioinformatics tools to predict functional impacts, with those receiving a consensus prediction of damaging effects becoming candidates for further analysis. The tools used were SIFT (2015) [22], PolyPhen-2 [23], MutationTaster 2 [24,25], PROVEAN (2015) [26], and Mutation Assessor (2015) [27].

rs77408762 showed suggestive evidence of association with both cholesterol and blood pressure measures in the WGS cohort. Under close sequence analysis, rs77408762 appeared to be part of a ten-base-pair sequence feature in the WGS group, and Sanger sequencing was performed to validate this observation.

### 2.2. Analysis of Norfolk Island ACOT4 Multi-Nucleotide Variant

#### 2.2.1. Sanger Sequencing

After identifying the ten-base-pair MNV in the WGS group, this sequence feature was confirmed in selected NIHS individuals (*n* = 80) by Sanger sequencing a 182 base-pair product that was generated using the primers 5′-GTATTGGAGGGGGCCTCTTG-3′ and 5′-GGACATGAGGAGAACCTGGG-3′ using BigDye^TM^ Terminator v3.1 Cycle Sequencing and an Applied Biosystems 3500 Bioanalyser (Thermo Fisher Scientific Inc., Waltham, MA, USA).

#### 2.2.2. Restriction Fragment Length Polymorphism (RFLP) Analysis

Once the sequence of the ten-base-pair MNV was confirmed by Sanger sequencing, RFLP analysis was performed to genotype it in the remaining NI cohort, using the restriction enzyme *Alu*I with recognition sequence AG|CT (New England Biolabs, Ipswich, MA, USA) and the same PCR primers as in the Sanger sequencing. Presence of the MNV eliminates the *Alu*I restriction site, allowing the genotype of the sample to be identified from restriction fragment number and length, determined by agarose gel electrophoresis.

### 2.3. Data Analysis—Norfolk Island

*ACOT4* MNV genotype data were tested for deviation from HWE with PLINK1.9 [21]. All other statistical analyses were performed with the R statistical programming language [28]. Linear or logistic multiple regression models were constructed for each trait as appropriate. The outcome variable was the phenotype and *ACOT4* genotype was the predictor variable, considering additive, genotype, and carrier models. Phenotypes investigated were waist circumference, blood glucose, blood cholesterol (total, HDL and LDL), blood triglycerides, and blood pressure (systolic and diastolic). The traits were modelled as continuous measures or dichotomised as follows in accordance with metabolic syndrome harmonised clinical diagnosis thresholds [29,30,31,32] and clinical reporting thresholds for total cholesterol [33].

• ‘large waist circumference’: ≥88 cm for females; ≥102 cm for males
• ‘low HDL’: <1.3 mmol/L for females; <1.0 mmol/L for males; or taking cholesterol medication
• ‘elevated BP’: systolic BP ≥130 mmHg OR diastolic BP ≥80 mmHg; or taking BP medication. Note that this criterion for metabolic syndrome diagnosis differs from the definition given in the AHA 2017 guidelines on high blood pressure [34] which state that both systolic AND diastolic BP should exceed these thresholds.
• ‘elevated blood triglycerides’: ≥1.7 mmol/L
• ‘elevated blood glucose’: ≥5.5 mmol/L
• ‘high total cholesterol’: ≥5.5 mmol/L; or taking cholesterol medication

Covariates included in multiple regression models were sex, age, and core pedigree membership as determined by Macgregor et al. [8]. In this part of the study, a significance threshold of *p* < 0.05 was used. Participants with missing genotypes or phenotypes were excluded from those analyses.

### 2.4. Data Analysis—UK Biobank

**Figure 1:**
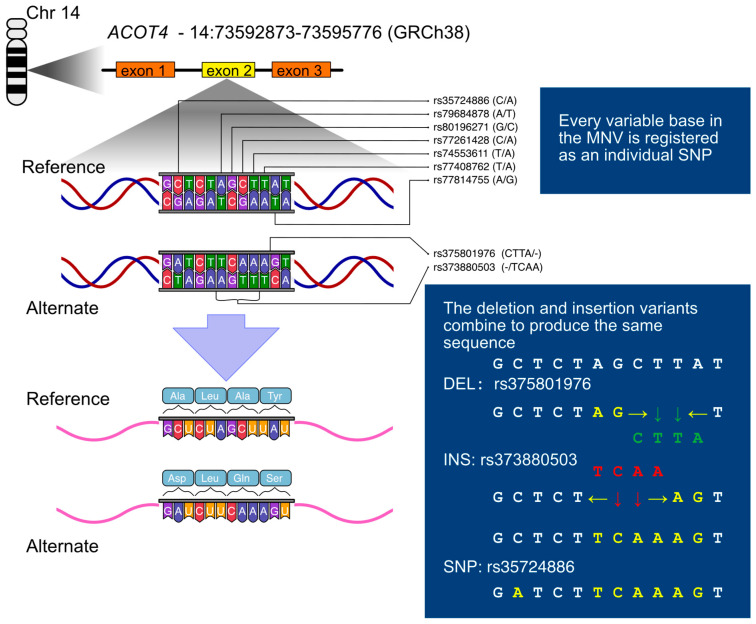
Illustration of the *ACOT4* ten-base-pair in-frame multi-nucleotide variant (MNV) and its translational consequences. The sequence of the MNV was determined by whole genome sequencing and Sanger sequencing in the Norfolk Island Health Study. It is annotated in the reference databases either as individual SNPs, or as a deletion and insertion, which in combination result in the same sequence. The MNV alters each of the four codons it lies across, with one synonymous and three missense mutations.

Genotypes consistent with the MNV were confirmed in the 2022 release of UKBB WES data [15]. The individual component SNPs of the main body of the MNV (14:73593808–73593813) were not annotated in these data. However, the SNP rs35724886 was recorded, as were the deletion (rs375801976) and insertion (rs776099821) variants, which result in the MNV sequence when combined (see Figure 1). Analysis of these three variants found no deviations from the expected combination of genotypes consistent with the MNV hypothesis. For simplicity, rs35724886 was used as a proxy for the MNV in further analysis. rs35724886 was tested for deviation from HWE with PLINK1.9.

Based on the results of the models tested in the NIHS, we used additive multiple regression models to test for association between the proxy SNP and BP (systolic and diastolic), total cholesterol, and HDL cholesterol. The traits were tested as continuous measures with linear regression and dichotomised according to the metabolic syndrome diagnostic criteria described above. All models included covariates age and sex. Blood pressure models also included total cholesterol, and the linear models included BP medication status as covariates (medication status is included in the dichotomous definition). Likewise, the cholesterol models included elevated BP, and the linear models included cholesterol medication status as covariates (again, medication status is included in the dichotomous definition). The number of effective tests was calculated to be five according to Galwey’s method [35], computed using the poolr (v1.2-0) package for R [36]. The significance threshold for trait association was set to *p* < 0.01 according to the conservative Bonferroni correction for multiple comparisons, using α = 0.05. Participants with missing genotypes or phenotypes were excluded.

Additionally, medication statuses for BP and cholesterol were modelled as outcome variables post hoc as an additional validation. We also tested for association between rs35724886 and stroke, as this was reported in a prior sequencing study [12]. Prior stroke status data came from the questionnaire records, and there was no differentiation between ischaemic and haemorrhagic sub-types in this dataset.

### 2.5. MNV In Silico Assessment

**Figure 2:**
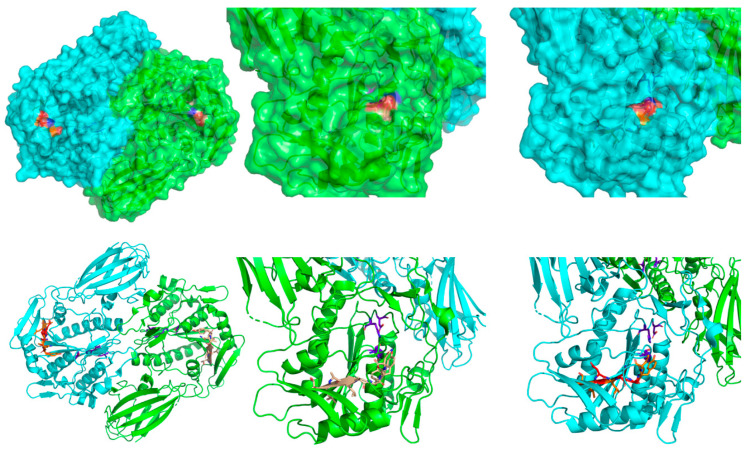
Modified structure of ACOT4 (PDB identifier 3k2i). The mature protein is a homodimer. Strand A (green) shows the reference sequence of the MNV (beige). Strand B (cyan) shows the alternative sequence (orange—conserved residues; red—substituted residues). The top images show surface rendering, while the bottom images show cartoon ribbon structure. The predicted active site is shown in both strands (purple). The variant sequence occurs at the transition between a β-sheet and loop region, which allows for some structural flexibility.

We used a suite of computational prediction tools to assess the effects of the amino acid changes encoded by the MNV, which are different from those encoded by the variants annotated in the reference databases. The tools used were DDMut 2023 [37], MutationTaster 2021 [25], SIFT [22], and PolyPhen2 [23]. DDMut calculates the global change in Gibbs free energy, Mutation Taster uses random forest models, SIFT compares homologous proteins, and PolyPhen2 uses a naïve Bayes classifier. DDMut and MutationTaster 2021 can compute predictions for multiple substitutions, such as that encoded by the MNV, while the other tools consider each change in isolation. We used the mutation modelling capabilities of PyMOL (v2.5.7) [38] to visualise the consequences of the MNV in the structure of ACOT4 (PDB identifier 3k2i) as shown in Figure 2.

### 2.6. Reporting

We have used the STREGA reporting guidelines in the preparation of this article [39].

## 3. Results

### 3.1. Identification of the ACOT4 Multi-Nucleotide Variant in Norfolk Island

WGS analysis of NIHS participants identified a ten-base-pair in-frame coding MNV in *ACOT4*, which was validated with Sanger sequencing and genotyped using an RFLP assay. This MNV results in three in-frame amino acid changes, which could potentially alter ACOT4 enzymatic function (see Figure 1).

The reference genome sequence at this locus (GRCh38 chr14:73593804–73593813) is 5′-CTCTAGCTTA-3′, while the MNV sequence is 5′-ATCTTCAAAG-3′, and should be annotated as NM_152331.4:c.560_569delinsATCTTCAAAG according to HGVS recommendations [40]. The MNV alters all four of the codons over which it lies, with one synonymous and three missense substitutions. The reference amino acid sequence at this position is Ala-Leu-Ala-Tyr, while the MNV sequence is Asp-Leu-Gln-Ser (p.Ala187_Tyr190delinsAspLeuGlnSer). There are no frame-shift effects from this variant.

### 3.2. The MNV in NIHS: Relationship with Blood Pressure and Cholesterol Levels

Eleven participant samples failed genotyping and were excluded from models. The minor allele frequency (MAF) of the *ACOT4* MNV was 21.3% in the Norfolk Island cohort, with no significant difference between core pedigree members (MAF = 21.8%) and non-members (20.6%). The MNV did not deviate from HWE in this cohort (*p* = 0.81). Cohort summary statistics for BP and cholesterol traits are presented in Table 1. The remaining trait summary statistics are presented in Supplementary Table S1. The mean age of participants was 51 years (±16).

Multiple regression models for blood pressure and cholesterol traits in the NIHS are presented in Table 2. The additive linear model indicated that the MNV was associated with higher total cholesterol, while the additive categorical model indicated an association between the MNV and lower blood pressure. The *ACOT4* MNV was not associated with waist circumference, blood glucose, or blood triglycerides (see Supplementary Table S2). The MNV was also not associated with total cholesterol exceeding the clinical threshold of 5.5 mmol/L as a binary trait.

The results presented as outlined in Table 2 and Supplementary Table S2 indicated associations of the *ACOT4* MNV with BP and cholesterol profiles in the NI population isolate. However, we wanted to further explore this in a larger and more general population. The presence of the MNV at the same frequency in both the NI core pedigree and non-core pedigree members suggested that it is not a NI-specific founder variant and hence could be investigated in larger, non-related datasets such as the UKBB, for which both genetic data and the equivalent metabolic health traits were available (Table 1).

### 3.3. The MNV in UKBB: Relationship with Blood Pressure and Cholesterol Levels

Fifteen participants were missing genotypes for rs35729886 and were excluded from further analyses. Summary statistics for blood pressure, HDL, and total cholesterol in the UKBB are presented in Table 1. The mean age of the subjects was 56 years (±8). There was evidence that rs35729886 genotypes deviate from HWE in this cohort, with more carriers present than expected (*p* = 8.0 × 10^−9^). Additive regression models of the rs35724886 genotype with covariates age, sex, and medication for the outcome trait (cholesterol or BP), found significant associations for BP and total cholesterol (Table 3), in the same directions as were observed in the NIHS participants (Table 2).

These models indicated lower systolic BP, diastolic BP (Std β = −0.008; *p* = 4.5 × 10^−4^), and lower incidence of elevated BP and low HDL. The variant was not associated with total cholesterol above 5.5 mmol/L.

The UKBB has in-depth data on prescribed medication for BP and cholesterol management, which we analysed post hoc as dependent variables after including them as covariates in the regression analyses of the primary outcomes of interest. In this cohort there were *n* = 40,745 (20.3%) participants taking BP medication and *n* = 33,784 (16.8%) participants taking cholesterol medication. Post hoc analysis of medication use showed evidence of lower incidence of both BP and cholesterol medication use was associated with rs35724886 (Table 4).

### 3.4. rs35724886 Is Not Associated with Stroke in the UKBB

Stroke data in the UKBB was from the assessment questionnaire, which asked whether a doctor had diagnosed the participant with heart attack, stroke, angina, or high blood pressure. There was no distinction drawn between ischaemic or haemorrhagic stroke. There were 2798 cases of stroke in the dataset. We did not find any association between rs35724886 and stroke in this cohort (OR: 1.01; 95% CI: 0.95, 1.08; *p* = 0.72).

### 3.5. In Silico Functional Analysis of ACOT4 MNV

Structural modelling of the MNV places it at the edge of a β-sheet region and unstructured loop and not in close proximity to the predicted active site (Figure 2). The computational tool DDMut calculated the amino acid changes to have a destabilising effect on the Gibbs free energy (ΔΔG = −3.82). The other computational tools used conformed in predicting damaging effects. Mutation Taster 2021 predicted the MNV to be ‘deleterious’. SIFT, which considers individual amino acid changes separately, predicted each one to affect the protein, and gave each substitution a score of 0.00. PolyPhen2, also considering individual amino acid changes, predicted each one to be ‘probably damaging’ and gave each one a score of 1.000. These tools use a variety of approaches to predict the effects of variation on proteins, as noted in Section 2, but a principal assumption is that major changes to protein structure are likely to be deleterious. Yet the associations we observe in these two study cohorts point to a beneficial effect, and the high frequency of this variant in European populations show that it is at least well tolerated.

## 4. Discussion

### 4.1. Role of ACOT4 in Lipid Metabolism

This sequencing study has uncovered a multi-nucleotide variant with relatively high frequency in European and European-admixed populations associated with improved measures of BP and cholesterol. ACOT4′s role in long and very-long chain fatty acid (VLCFA) β-oxidation in peroxisomes has implications for cholesterol breakdown and biosynthesis, and the observed patterns in the NIHS and UKBB cohorts suggest that cholesterol levels are influenced by the change in ACOT4′s activity caused by this MNV, possibly by increasing the HDL component. Of note is that while increased total cholesterol was associated with this variant, total cholesterol levels exceeding the clinical threshold of 5.5 mmol/L were not.

Peroxisomes specialise in the β-oxidation of VLCFAs and require coenzyme A (CoA) in a key role; forming and degrading acyl-CoAs, mediated by Acyl-CoA Synthetases and Acyl-CoA Thioesterases [41]. Disruption to core lipid metabolic pathways is known to significantly contribute to metabolic conditions [42]. ACOT4, localised to peroxisomes, is a type I ACOT with enzymatic activity across a wide range of acyl-CoA lengths, cleaving acyl CoAs to produce free fatty acids (FFA) and CoASH [43,44]. Its highest affinity is for succinyl-CoA (four carbons), with varying activity on saturated FAs (eight carbons to twenty carbons) and unsaturated FAs (18:1, 18:2, 20:4) [43]. Peroxisomal β-oxidation does not fully consume FAs, with the final short FFAs exported to mitochondria for final β-oxidation into CO_2_ and H_2_O. Substrates that are oxidised in peroxisomes include the bile-acid intermediates di- and tri-hydroxycholestanoic acid (DHCA and THCA), which are breakdown products of cholesterol, and the terminal β-oxidation products include acetyl-CoA, which is a precursor molecule for cholesterol biosynthesis in the endoplasmic reticulum [45,46]. The precise role of *ACOT4* in this cholesterol-adjacent metabolic pathway is still not well understood, but the results we have presented here show that perturbations to this protein could have clinically relevant effects on circulating cholesterol balances. Blood pressure is affected by cholesterol levels and composition, with low HDL levels contributing to high BP. Thus, the association we see in both cohorts between the *ACOT4* MNV and improved BP outcomes is plausible and suggests that the close interrelationship between BP and cholesterol is driving this association.

When we tested post hoc for association between the MNV and medication use for BP and cholesterol management, we found corroborating evidence for the associations observed between the MNV and the physical measures. We would expect a true relationship to be observed in the clinic, and this seems borne out by the rates of medication prescribed being lower for individuals carrying the MNV.

### 4.2. Clinical Relevance of the MNV

While blood pressure is strongly affected by cholesterol, it can be high even when cholesterol levels are healthy and must be considered as a risk factor that is separate but not completely independent from cholesterol in heart disease [1]. The associations we observed here indicated a 3–4% reduction in elevated BP and low HDL. We did observe an increase in total cholesterol, but not in excess of the clinical threshold of 5.5 mmol/L. Given the role of dyslipidaemia in the aetiology of CVD including stroke, we might expect that this variant reduces stroke risk, rather than raising it. However, as our in silico analysis of this protein demonstrates, it is still reasonable to predict that this disruption in an important lipid metabolism pathway may have adverse effects on these conditions, highlighting the need for careful functional studies of these mechanisms.

The tagging SNP rs35724886 was reported to be associated with ischaemic stroke in the NHLBI exome sequencing project (OR: 2.04; *p* = 1.2 × 10^−7^) [12]. The investigation was conducted across several American study cohorts, with European ancestry and African ancestry individuals included. We note that the MAF reported in that study was 1.7% in the European ancestry group, and 18% in the African ancestry group, while the currently reported allele frequencies for these populations in the gnomAD reference are 21% and 3%, respectively. We found no evidence that rs35724886, and by extension the MNV, was associated with stroke in the UKBB, although it does not differentiate between ischaemic and haemorrhagic subtypes.

## 5. Conclusions

*ACOT4*’s precise role in metabolic health and disease remains incompletely characterised. However, rs35724886, which is a marker for the MNV, has been associated with key metabolite features in metabolomic studies [13], and *ACOT4* has also been proposed as a therapeutic target in non-alcoholic fatty liver disease [14]. This present study contributes to this growing body of evidence that *ACOT4* has a critical role in cardiovascular health and identifies rs35724886 as a label for a larger coding sequence feature of which it is a part. This ten-base-pair in-frame multi-nucleotide variant has clinically observable associations with desirable blood pressure and cholesterol measures, which are borne out by corresponding patterns of medication use to manage these conditions. However, the molecular mechanisms of these effects are still not clear and functional studies are needed to fully understand this gene and variant’s role in these metabolic processes.

## Figures and Tables

**Table 1:** Summary statistics of blood pressure, cholesterol, and HDL in Norfolk Island Health Study and UK Biobank.

Trait	NIHS *n* = 601 Females *n* = 340 (57%)	UKBB *n* = 200,625 Females *n* = 110,432 (55%)
Systolic BP in mmHg, mean (sd) [*n* = NA]	129.0 (24.1) [8]	137.7 (18.6) [213]
Diastolic BP in mmHg, mean (sd) [*n* = NA]	76.8 (13.4) [8]	82.2 (10.1) [213]
Elevated BP ^a^, *n* (%) [*n* = NA]	290 (50%) [8]	150,301 (75%) [148]
Total Cholesterol in mmol/L, mean (sd) [*n* = NA]	5.64 (1.12) [2]	5.70 (1.14) [9682]
High Cholesterol ^b^, *n* (%) [*n* = NA]	306 (51%) [2]	133,751 (66%) [9682]
HDL Cholesterol in mmol/L, mean (sd) [*n* = NA]	1.40 (0.36) [6]	1.46 (0.38) [25,032]
Low HDL Cholesterol ^c^, *n* (%) [*n* = NA]	145 (24%) [6]	59,803 (30%) [25,032]

^a.^ Elevated BP; systolic BP ≥ 130 mmHg or diastolic BP ≥ 80 mmHg or blood pressure medication. ^b.^ High cholesterol; ≥5.5 mmol/L or cholesterol medication. ^c.^ Low HDL; <1.3 mmol/L for females, <1.0 mmol/L for males or cholesterol medication.

**Table 2:** *ACOT4* MNV additive, genotype, and carrier model summaries for blood pressure, HDL, and total cholesterol in the Norfolk Island Health Study.

Model	OR	95% CI	*p*		Raw β	Std β	95% CI	*p*
	Elevated blood pressure				Systolic blood pressure			
Additive	0.70	0.51, 0.97	0.032 *		−2.164	−0.052	−4.968, 0.641	0.13
Heterozygous	0.90	0.60, 1.33	0.58		−1.163	−0.023	−4.608, 2.281	0.51
Homozygous	0.20	0.05, 0.57	0.0055 *		−7.211	−0.061	−15.254, 0.832	0.079
MNV carrier	0.78	0.53, 1.13	0.19		−1.843	−0.037	−5.163,1.478	0.28
WT carrier	4.84	1.70, 17.39	0.0064 *		6.799	0.058	−1.147, 14.744	0.093
	Low HDL				HDL cholesterol			
Additive	1.24	0.88, 1.72	0.21		−0.002	−0.004	−0.051, 0.046	0.92
Heterozygous	0.93	0.60, 1.42	0.74		0.019	0.026	−0.040, 0.079	0.52
Homozygous	2.95	1.22, 7.07	0.015 *		−0.072	−0.040	−0.214, 0.070	0.32
MNV carrier	1.08	0.72, 1.61	0.70		0.010	0.013	−0.048, 0.067	0.74
WT carrier	0.33	0.14, 0.79	0.012 *		0.079	0.044	−0.061, 0.220	0.27
	High cholesterol				Total cholesterol			
Additive	1.18	0.88, 1.59	0.26		0.161	0.082	0.010, 0.311	0.037 *
Heterozygous	1.17	0.82, 1.68	0.39		0.213	0.090	0.029, 0.398	0.023 *
Homozygous	1.44	0.62, 3.40	0.39		0.166	0.030	−0.269, 0.601	0.45
MNV carrier	1.20	0.85, 1.70	0.31		0.208	0.090	0.031, 0.386	0.022 *
WT carrier	0.73	0.31, 1.70	0.47		−0.089	−0.016	−0.521, 0.342	0.68

Binary traits were modelled with multiple logistic regression (left columns). Continuous traits were modelled with multiple linear regression (right columns). Systolic blood pressure was measured in mmHg. HDL and total cholesterol were measured in mmol/L. Elevated blood pressure is defined as SBP ≥ 130 mmHg or DBP ≥ 80 mmHg, or taking blood pressure medication. Low HDL is defined as HDL < 1.3 mmol/L for females or HDL < 1.0 mmol/L for males, or taking cholesterol medication. High cholesterol is defined as total cholesterol ≥ 5.5 mmol/L, or taking cholesterol medication. All models included age, sex, and NIHS core pedigree membership as covariates. Blood pressure models additionally included total cholesterol. Cholesterol models additionally included elevated blood pressure. Significance (*) was set at *p* < 0.05.

**Table 3:** *ACOT4* rs35724886 additive model summaries for blood pressure, HDL, and total cholesterol in the UK Biobank.

OR	95% CI	*p*		Raw β	Std β	95% CI	*p*
Elevated blood pressure				Systolic blood pressure			
0.96	0.95, 0.98	0.00014 *		−0.196	−0.006	−0.330, −0.062	0.0041 *
Low HDL				HDL cholesterol			
0.97	0.95, 0.99	0.00026 *		0.002	0.002	−0.001, 0.004	0.28
High cholesterol				Total cholesterol			
1.00	0.99, 1.02	0.42		0.011	0.005	0.003, 0.018	0.0098 *

Binary traits were modelled with multiple logistic regression (left). Continuous traits were modelled with multiple linear regression (right). Systolic blood pressure was measured in mmHg. HDL and total cholesterol were measured in mmol/L. Elevated blood pressure is defined as SBP ≥ 130 mmHg or DBP ≥ 80 mmHg or taking prescribed blood pressure medication. Low HDL is defined as HDL < 1.3 mmol/L for females, or HDL < 1.0 mmol/L for males, or taking medication for cholesterol management. High cholesterol is defined as total cholesterol ≥ 5.5 mmol/L for both sexes, or taking medication for cholesterol management. All models included age and sex as covariates. Blood pressure models additionally included total cholesterol, and linear models included BP medication status. Cholesterol models additionally included elevated blood pressure, and linear cholesterol models corrected for cholesterol medication status. Significance (*) was set at *p* < 0.01.

**Table 4:** *ACOT4* rs35724886 genotype counts by blood pressure and cholesterol medication use, with additive, genotype, and carrier regression model summaries.

	Blood Pressure Medication Use					Cholesterol Medication Use			
Genotype	BP Meds		No BP Meds			Chol Meds		No Chol Meds	
Wild Type	25,833 (20.5%)		100,433 (79.5%)			21,311 (16.9%)		104,955 (82.1%)	
Heterozygous	13,144 (20.1%)		52,094 (79.9%)			10,995 (16.8%)		54,243 (83.1%)	
Homozygous	1768 (19.4%)		7338 (80.6%)			1478 (16.3%)		7628 (83.7%)	
Model	OR	95% CI		*p*		OR	95% CI		*p*
Additive	0.95	0.93, 0.97		0.0000018 *		0.97	0.95, 0.99		0.0020 *

Medication use was modelled with logistic regression, with covariates sex and age. Significance (*) was set at *p* < 0.01. These results were calculated post hoc.

## Data Availability

All data generated or analysed during this study are included in this published article.

## Supplementary Materials

The following supporting information can be downloaded at: https://www.mdpi.com/article/10.3390/genes17020205/s1, Table S1. Summary statistics of traits not reported in the main text for the NIHS and UKBB. Table S2. Norfolk Island Health Study regression results not reported in the main text.
