# Supplementary material for: An ACOT4 Multi-Nucleotide Variant Is Associated with Cardiovascular Risk in Norfolk Island and UK Biobank Cohorts
Source: Genes (Basel). 2026 Feb 9;17(2):205. doi: 10.3390/genes17020205 (PMC12940796; doi:10.3390/genes17020205)
Supplement: Supplementary file 1 [file genes-17-00205-s001.zip › genes-4068563-supplementary.pdf]

Supplementary tables for "An ACOT4 Multi-Nucleotide Variant is Associated with Cardiovascular Risk in Norfolk Island and UK Biobank Cohorts"

1 Trait summary statistics

Supplementary Table S1: Summary statistics of traits not reported in the main text for the NIHS and UKBB.

Trait	NIHS n = 601<br>females n = 340 (57%)
Waist circumference in cm, by sex, mean (sd) [n = NA]	female 82 (12) [17]<br>male 95 (11) [17]
Large waist circumference, by sex, n (%) [n = NA]	female 99 (29%) [17]<br>male 60 (23%) [17]
Blood glucose in mmol/L, mean (sd) [n = NA]	5.64 (1.01) [27]
Elevated blood glucose, n (%) [n = NA]	292 (49%) [27]
LDL Cholesterol in mmol/L, mean (sd) [n = NA]	2.84 (0.98) [4]
High LDL Cholesterol, n (%) [n = NA]	479 (80%) [4]
Triglycerides in mmol/L, mean (sd) [n = NA]	1.96 (1.24) [2]
High triglycerides, n (%) [n = NA]	302 (50%) [2]
Diastolic BP in mmHg, mean (sd) [NA]	76.8 (13.4) [8]

2 Multiple regression models in NIHS

Supplementary Table S2: Norfolk Island Health Study regression results not reported in the main text.

Model	OR	95% CI	P	Raw β	Std β	95% CI	P
	Large WC: ≥ 88 cm (sex = F)<br>≥ 102 cm (sex = M)			Waist circumference (cm)			
Additive	0.85	0.60, 1.19	0.36	-0.29	-0.013	-1.88, 1.30	0.72
Heterozygous	0.80	0.53, 1.20	0.28	-0.60	-0.022	-2.55, 1.35	0.54
Homozygous	0.90	0.31, 2.29	0.83	0.34	0.0052	-4.24, 4.91	0.88
MNV carrier	0.81	0.54, 1.20	0.29	-0.50	-0.018	-2.38, 1.38	0.60
WT carrier	1.03	0.41, 2.97	0.95	-0.55	-0.0084	-5.07, 3.97	0.81

	Elevated glucose ≥ 5.5 mmol/L			Blood glucose (mmol/L)			
Additive	1.02	0.76, 1.36	0.92	-0.038	-0.022	0.18, 0.10	0.60
Heterozygous	1.00	0.70,1.43	0.99	-0.015	-0.0070	-0.19, 0.16	0.87
Homozygous	1.08	0.47, 2.49	0.85	-0.14	-0.029	-0.54, 0.26	0.49
MNV carrier	1.01	0.71, 1.43	0.97	-0.029	-0.014	-0.20, 0.14	0.73
WT carrier	0.92	0.41, 2.12	0.85	0.14	0.028	-0.26, 0.53	0.50
	High LDL ≥ 2.0 mmol/L			LDL cholesterol (mmol/L)			
Additive	1.22	0.84, 1.79	0.31	0.103	0.060	-0.031, 0.236	0.13
Heterozygous	1.34	0.84, 2.18	0.23	0.172	0.083	0.008, 0.336	0.039 *
Homozygous	1.16	0.45, 3.43	0.77	0.003	0.001	-0.382, 0.387	0.99
MNV carrier	1.31	0.84, 2.08	0.24	0.15	0.076	-0.004, 0.311	0.057
WT carrier	0.95	0.33, 2.43	0.92	0.058	0.012	-0.323, 0.440	0.76
	Elevated trig ≥1.7 mmol/L			Triglycerides (mmol/L)			
Additive	1.11	0.82, 1.49	0.50	0.015	0.007	-0.152, 0.182	0.86
Heterozygous	1.33	0.92, 1.92	0.13	0.050	0.020	-0.154, 0.255	0.63
Homozygous	0.71	0.28, 1.71	0.46	-0.073	-0.012	-0.556, 0.410	0.77
MNV carrier	1.24	0.87, 1.77	0.23	0.037	0.015	-0.161, 0.234	0.71
WT carrier	1.55	0.66, 3.97	0.33	0.091	0.015	-0.386, 0.568	0.71
				Diastolic BP (mmHg)			
Additive				-0.67	-0.029	-2.41, 1.07	0.45
Heterozygous				0.63	0.023	-1.50, 2.76	0.56
Homozygous				-5.086	-0.078	-10.065, -0.106	0.045 *

MNV carrier	-0.013	-0.0005	-2.073, 2.048	0.99
WT carrier	5.31	0.081	0.391, 10.228	0.034 *

Binary traits were modelled with multiple logistic regression (left columns). Continuous traits were modelled with multiple linear regression (right columns). All models included age, sex, and NIHS core pedigree membership as covariates. Blood pressure models additionally included total cholesterol. Cholesterol models additionally included elevated blood pressure. Significance (*) was set at $p < 0.05$.
